# Comparative study of *nosZ*I and *nosZ*II clade isolates: insights into their responses to environmental variables and soil fertilization types

**DOI:** 10.3389/fpls.2025.1537010

**Published:** 2025-06-06

**Authors:** Haoran Li, Qishi Luo, Lichao Sun, Huicui Xu, Xiaodong Hao, Kai Zhu, Ming Li, Bao Li, Wei Jiao, Jibiao Geng, Zhiqun Chen, Lihua Huang, Zongwei Xia

**Affiliations:** ^1^ Shandong Provincial Key Laboratory of Water and Soil Conservation and Environmental Protection, College of Resources and Environment, Linyi University, Linyi, China; ^2^ Linyi Ecological Environmental Bureau, Linyi, China

**Keywords:** nitrous oxide emissions, *nosZ* clade I, *nosZ* clade II, nitrous oxide reduction, environmental factors, fertilizer types

## Abstract

**Introduction:**

Soil nitrous oxide (N_2_O) emissions are a major contributor to global warming and climate change. Microbial N_2_O reduction via the *nosZ* gene—classified into clade I and clade II—is the only known biological sink for N_2_O.

**Methods:**

In this study, we isolated two N_2_O-reducing bacterial strains: an *Enterobacter* sp. harboring *nosZ* clade I (*nosZ*I) and a *Pseudomonas* sp. harboring *nosZ* clade II (*nosZ*II). We evaluated their performance under different environmental conditions and their effects on N_2_O emissions from agricultural soils subjected to varying fertilization strategies.

**Results:**

Pure culture experiments revealed that growth and N_2_O-reducing activity in both strains were significantly influenced by pH, oxygen concentration, nitrate levels, and carbon source. Notably, the *nosZ*II strain demonstrated broader environmental adaptability and higher N_2_O-reduction efficiency across a range of conditions. In soil microcosm experiments, N_2_O emissions were strongly affected by fertilization type, with mixed organic-inorganic treatments producing the highest emissions. Inoculation with the *nosZ*II strain significantly reduced N_2_O emissions in soils receiving inorganic or combined fertilizers. In contrast, the *nosZ*I strain tended to increase emissions, except under the mixed fertilization regime.

**Discussion:**

These findings highlight the importance of selecting N_2_O-reducing microbial strains based on their functional capacity and environmental tolerance. This work advances the development of targeted microbial strategies for mitigating N_2_O emissions, supporting more sustainable and climate-resilient agricultural practices.

## 1 Introduction

Nitrous oxide (N_2_O) is a potent greenhouse gas that significantly contributes to global warming and is currently the leading cause of stratospheric ozone depletion (Ravishankara et al., 2009; World Meteorological Organization, 2022). Agricultural soils represent a major source of N_2_O emissions, with nitrogen fertilizer applications accounting for approximately 53% of global anthropogenic N_2_O emissions (Tian et al., 2020). Driven by global population growth and intensified land use, the increasing application of nitrogen fertilizers in agriculture has led to a substantial rise in N_2_O emissions (Bahram et al., 2022). Thus, developing effective strategies to mitigate N_2_O emissions while sustaining crop productivity and environmental health is crucial, especially under mounting pressure on the global food supply system.

Although soil amendments—such as biochar and biological inhibitors—have been explored for reducing N_2_O emissions, their effectiveness remains inconsistent and context-dependent. For instance, the efficacy of biochar varies widely depending on factors such as feedstock type, soil properties, climatic conditions, and management practices, with some studies reporting neutral or even adverse effects on greenhouse gas emissions (Sanford et al., 2012; Jones et al., 2013; Zhang et al., 2020a). Likewise, biological inhibitors, including urease inhibitors (UIs) and nitrification inhibitors (NIs) like dicyandiamide (DCD) and 3,4-dimethylpyrazole phosphate (DMPP), have shown variable performance and may negatively affect microbial biomass or plant health when overapplied (Carneiro et al., 2010; Suenaga et al., 2019; Maheshwari et al., 2023). Moreover, recent studies have questioned the effectiveness of DCD in reducing N_2_O emissions relative to conventional urea-based fertilization (Maheshwari et al., 2023). There is a clear need for alternative mitigation strategies that are both effective and environmentally sustainable.

A promising biological approach lies in harnessing the activity of nitrous oxide reductase (Nos), the only known enzyme capable of reducing N_2_O to dinitrogen (N_2_), thereby serving as the sole biological sink for N_2_O in the nitrogen cycle (Conthe et al., 2018). The *nosZ* gene, encoding this enzyme, is categorized into two phylogenetically distinct clades—clade I and clade II—which differ in their genetic makeup, physiological traits, and ecological functions (Jones et al., 2013; Domeignoz-Horta et al., 2016; Hallin et al., 2018). Genomic analyses have identified the taxonomic affiliations of these clades: *nosZ* clade I is primarily found in genera such as *Bradyrhizobium*, *Pseudomonas*, *Paracoccus*, *Ralstonia*, *Thiobacillus*, and *Rhodopseudomonas*, while *nosZ* clade II is more widely distributed across diverse genera including *Anaeromyxobacter*, *Gemmatimonas*, *Opitutus*, and *Hydrogenobacter* (Jones et al., 2013; Graf et al., 2014; Orellana et al., 2014; Domeignoz-Horta et al., 2016; Avşar and Aras, 2020). Clade II bacteria, in particular, have been observed to dominate in systems where N_2_O serves as the primary electron acceptor, whereas clade I bacteria are more abundant under nitrate-rich conditions (Suenaga et al., 2019).

The abundance and functional efficiency of *nosZ*-carrying microorganisms are influenced by a range of environmental variables, including pH, oxygen availability, carbon sources, and nitrate concentrations (Liu et al., 2010; Lu and Chandran, 2010; Desloover et al., 2014; Park et al., 2017; Suenaga et al., 2018; Qi et al., 2022; Shaaban et al., 2023). Despite advances in understanding the phylogenetic and functional diversity of *nosZ* communities, there remains a significant knowledge gap regarding how *nosZ* clade I and II bacteria compare in their N_2_O reduction capacities under different fertilization regimes and environmental conditions in agricultural soils.

Given their distinct genetic and ecological characteristics, we hypothesize that: (1) *nosZ* clade II bacteria exhibit broader environmental tolerance—particularly to variations in pH, oxygen, nitrate, and carbon sources—compared to *nosZ* clade I bacteria; and (2) the N_2_O-reducing performance of *nosZ* clade I and II bacteria will differ across soils managed with different fertilization strategies (e.g., inorganic, organic, or mixed). To test these hypotheses, we isolated and characterized *nosZ* clade I and II bacteria from fertilized agricultural soils using nitrogen-limited, N_2_O-enriched media under anaerobic conditions. We conducted both pure culture experiments and soil microcosm trials to assess the growth and N_2_O reduction efficiency of each clade. The objectives of this study are: (1) to compare the growth responses and N_2_O reduction activity of *nosZ* clade I and II isolates under a range of environmental conditions relevant to agricultural soils; (2) to evaluate the effects of these isolates on soil N_2_O emissions under different fertilization strategies.

## 2 Materials and methods

### 2.1 Study site and soil sample collection

Soil samples were collected from a controlled-environment greenhouse at Linyi University (35.119762° N, 118.28975° E), Linyi, China. The field was cultivated with cucumber (Cucumis sativus L. cv. ‘Zhongnong 62’) and pepper (Capsicum annuum L. cv. ‘Shifeng 802’). The basic physicochemical characteristics of the soil are summarized in **Table 1**. Topsoil (0–15 cm) was collected during the non-growing season (totaling 10 kg), homogenized, sieved through a 2 mm mesh, and partitioned into 300 g for bacterial screening and 8 kg for soil incubation experiments.

**Table 1:** The basic physicochemical characteristics of soil ^1^.

Bulk density (g/cm³)	WFPS ^2^ (%)	pH	EC ^2^ (us/cm)	Organic matter (g/kg)	Total nitrogen (g/kg)	Available phosphorus (mg/kg)	Available potassium (mg/kg)
1.45	41%	5.9	471	15.72	1.053	37.57	2

^1^Sampling was conducted on August 11, 2024, at a controlled-environment greenhouse located at Linyi University (35.119762° N, 118.28975° E), Linyi, Shandong Province, China.

^2^WFPS and EC represent soil water-filled pore space and electrical conductivity, respectively.

### 2.2 Isolation, growth, and N_2_O reduction capacity of isolates

Two isolates were selected for this study: one harboring the *nosZ* clade I gene (hereafter referred to as the *nosZ*I strain) and the other harboring the *nosZ* clade II gene (*nosZ*II strain). Morphologically, both isolates formed circular, smooth, mucoid colonies. The *nosZ*I strain produced opaque, white colonies, whereas the *nosZ*II strain formed translucent, creamy-white colonies.

Isolation Procedure: Sterilized Schott bottles were filled with dried soil and sterile water, then pre-incubated anaerobically for 7 days. The soil suspension was homogenized with sterile glass beads, transferred to fresh Schott bottles, amended with sodium acetate anhydrous, flushed with high-purity helium, and spiked with N_2_O to a final concentration of 8%. Cultures were incubated at 30°C with shaking at 180 rpm. Helium and N_2_O were replenished every 2 days. After 7 days, samples were serially diluted and plated on nitrogen-free medium, then transferred to a helium-flushed, sealed incubation chamber supplemented with N_2_O and incubated anaerobically for 48 hours at 30°C. Distinct colonies were subcultured, and genomic DNA was extracted for PCR-based identification of *nosZ* clade I and II genes using specific primers. Two isolates showing strong N_2_O reduction capacity were selected for further study (see **Supplementary Material** for full protocols).

Experimental Conditions: The effects of environmental factors on growth and N_2_O reduction were assessed under varying conditions of pH (4, 6, 7, 8, 10), oxygen (0%, 0.1%, 1%, 5%, 21% v/v), nitrate (0, 5, 50, 100, 500 mg N L^-^¹), and carbon source (sodium acetate, glycerol, disodium succinate, glucose, and hydroxyethyl cellulose). Each treatment was replicated three times.

Culture Medium and Setup: Tryptic Soy Broth (TSB; Land Bridge Co., China) was prepared by dissolving 30 g in 1 L of distilled water. The formulation included: tryptic peptone (17.0 g), plant-derived peptone (3.0 g), NaCl (5.0 g), K_2_HPO_4_ (2.5 g), and glucose (2.5 g). After sterilization, 20 mL of TSB was added to 500 mL Schott bottles. The pH was adjusted to one of five levels (4–10), and the medium was inoculated with 1% (v/v) bacterial culture and 1.5 mL of 99.9% N_2_O. Bottles were incubated at 30°C and 180 rpm for 10 hours.

After incubation, 30 mL of headspace gas was withdrawn using a syringe and transferred to pre-evacuated vials for N_2_O measurement via gas chromatography (Agilent 7890A, USA). Optical density at 600 nm (OD_600_) was measured immediately afterward.

In O_2_ and NO_3_^-^ experiments, pH was held constant at 7, while O_2_ or nitrate concentrations were varied. For carbon source treatments, an inorganic salt base medium (carbon-free) was used, supplemented with a carbon source providing 50 mg of carbon, and pH was maintained at 7.

### 2.3 Soil microcosm experiment

The microcosm experiment consisted of 12 treatments (described in **Table 2**), each with three replicates and 12 sampling intervals for N_2_O analysis. Treatments included: CK (no fertilizer or bacteria), IF (inorganic fertilizer), OF (organic fertilizer), and MF (70% organic + 30% inorganic fertilizer). Fertilizer application was standardized at 2.5 mg N g^-^¹ dry soil. The inorganic fertilizer was a compound formula (15% N–P_2_O_5_–K_2_O; Stanley Co., China), and the organic fertilizer was sheep manure compost (1.72% N, 2.24% P_2_O_5_, 2.42% K_2_O; JiYa Co., China). Treatments suffixed with ‘*nosZ*I’ or ‘*nosZ*II’ indicate inoculation with the respective strain at a concentration of 10^9^ CFU per bottle (based on standard plate count method).

**Table 2:** Experimental treatment of soil microcosm ^1^.

Treatment	Fertilizer types	Bacterial agents
CK	No-fertilizer	Sterile water
CK*nosZ*I	No-fertilizer	*nosZ*I-strain
CK*nosZ*II	No-fertilizer	*nosZ*II-strain
IF	Inorganic fertilizer	Sterile water
IF*nosZ*I	Inorganic fertilizer	*nosZ*I-strain
IF*nosZ*II	Inorganic fertilizer	*nosZ*II-strain
OF	Organic fertilizer	Sterile water
OF*nosZ*I	Organic fertilizer	*nosZ*I-strain
OF*nosZ*II	Organic fertilizer	*nosZ*II-strain
MF	Mixed fertilizer	Sterile water
MF*nosZ*I	Mixed fertilizer	*nosZ*I-strain
MF*nosZ*II	Mixed fertilizer	*nosZ*II-strain

^1^The experiment was conducted from September 20 to October 12, 2024, at the lab, Linyi University, Linyi City, Shandong Province, China.

The following is the detailed procedure of the plate counting method: 1 mL of bacterial suspension was inoculated into 100 mL of TSB liquid medium and incubated at 30 ± 0.5°C with shaking (180 rpm) for 12 h. Subsequently, serial dilutions were performed to achieve a 10^-6^ gradient. Aliquots (10 μL) of 10^-4^, 10^-5^, and 10^-6^ dilutions were spread onto nitrogen-free agar plates and incubated at 30 ± 0.5°C for 24 h. Colonies within the valid range (30–300 CFU/plate) were enumerated, and the original cell concentration (N, CFU/mL) was calculated using the formula:


$$N=\frac{C}{D\times V}$$

Where: C is the colony count (CFU); D is the dilution factor (10^-n^); V is the inoculation volume (mL).

For the *nosZ*I strain, the valid dilution (colony count C=203, dilution factor D=10^-5^, inoculation volume V=0.01 mL) yielded a calculated cell concentration of 2.03×10^8^ CFU/mL. Similarly, for the *nosZ*II strain (C=246, D=10^-5^, V=0.01 mL), the concentration was determined as 2.46×10^8^ CFU/mL. To achieve the target inoculum density of 1×10^9^ CFU/mL, the required volumes were adjusted to 5 mL for *nosZ*I and 4 mL for *nosZ*II, ensuring standardized inoculation across experimental replicates.

Microcosm Setup: A total of 150 g of soil was placed into 500 mL Schott bottles. Fertilizer was thoroughly mixed into the soil, and 50 g of untreated soil was layered on top. Bacterial cultures and sterile water (total 50 mL) were sprayed onto the surface to adjust moisture to 20%. Control treatments received sterile water only. Bottles were incubated at 26°C in darkness, with soil moisture maintained at 20% via periodic weighing and replenishment with sterile water. The sterile water used was deionized water, with a pH of 7 and a resistivity of 18.2 MΩ·cm.

### 2.4 Gas sampling and N_2_O measurement

Gas samples were collected at 6 h, and 1, 2, 3, 4, 5, 7, 9, 12, 15, 18, and 21 days post-treatment. Prior to each sampling, bottles were flushed with ambient air and sealed with gas-tight lids equipped with a three-way valve. At each interval, 30 mL of headspace gas was sampled and stored in pre-evacuated vials. During each sampling, gas was collected at 0, 1, 2, and 3 hours to determine emission rates. N_2_O concentrations were measured using gas chromatography (Agilent 7890A, USA).

The formula for calculating the soil N_2_O emission rate (*F*) is as follows:


$$\text{F}=\frac{{\text{ρ}}_{N_{2}O}\times \text{V}\times 273}{\left(273+\text{T}\right)\times \text{m}}\times \frac{{\text{d}}_{\text{c}}}{{\text{d}}_{\text{t}}}$$

Where: $\rho_{{\text{N}}_{2}\text{O}}$ represents the density of N_2_O-N under standard conditions (1.25 g L^-^¹); $\frac{d_{c}}{d_{t}}$ represents the rate of change in N_2_O concentration in the bottle (ppm h^-^¹); *T* is the incubation temperature (C); *V* is the volume of the incubation bottle (m³); *m* is the dry weight of the incubated soil (kg).

The formula for calculating the cumulative soil N_2_O emissions (*E*) is as follows:


$$\text{E}=\sum \frac{\left({\text{F}}_{\text{i}+1}+{\text{F}}_{\text{i}}\right)}{2}\times ({\text{T}}_{\text{i}+1}-{\text{T}}_{\text{i}})\times 24$$

Where: $F_{i+1}$ is the average emission flux of the gas collected during the i+1 sampling (mg g^-^¹ h^-^¹); $F_{i}$ is the average emission flux of the gas collected during the i sampling (mg g^-^¹ h^-^¹); $T_{i+1}-T_{i}$ is the time interval between the i+1 and i gas sampling events (days).

### 2.5 Statistical analysis

In the pure culture experiments, one-way ANOVA followed by Duncan’s multiple range test was used to assess differences in OD_600_ and N_2_O reduction efficiency across treatments. Homogeneity of variance was tested using Levene’s test; data were log-transformed if necessary. Statistical significance was set at p < 0.05. Pearson correlation analysis was used to explore relationships between environmental factors, bacterial growth, and N_2_O reduction, with p < 0.05 considered significant.

In the soil microcosm experiment, two-way ANOVA was performed to assess interactions between fertilizer type and *nosZ* genotype on N_2_O emissions. Repeated measures ANOVA, combined with the least significant difference (LSD) test, was used to analyze temporal trends. One-way ANOVA with Duncan’s test was used to compare emissions among treatments at each time point. Homogeneity of variance was again checked with Levene’s test, and log transformations were applied as needed.

All statistical analyses were performed using SPSS v21.0 (SPSS Inc., Chicago, IL, USA). Figures were generated using Origin 2022 (OriginLab Corp., Northampton, MA, USA).

## 3 Results

### 3.1 Growth and N_2_O-reducing capacity of isolates under different environmental conditions

**Figure 1:**
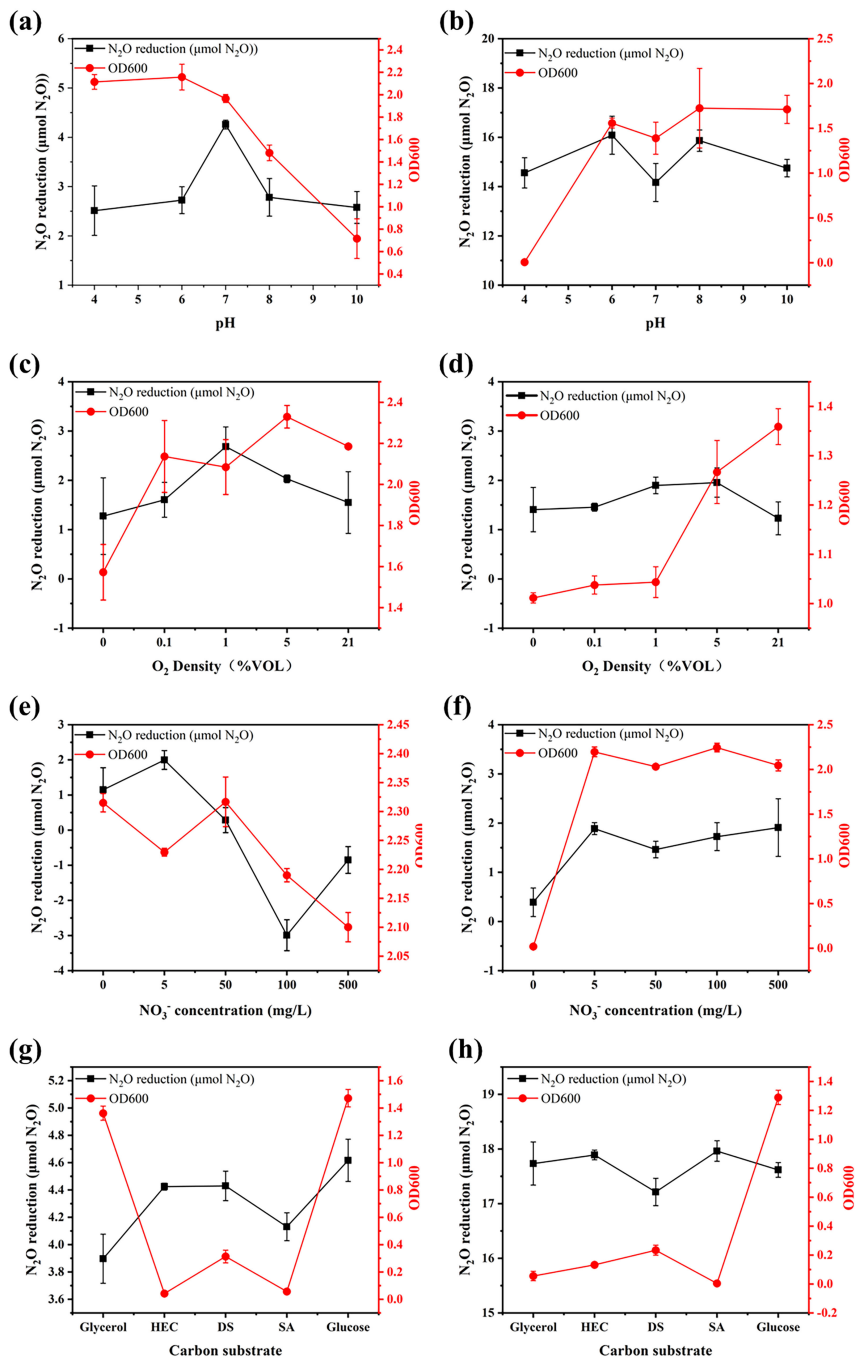
The growth (OD600) and N_2_O-reducing activity of *nosZ*I **(a, c, e, g)** and *nosZ*II **(b, d, f, h)** strains responding to environmental factors through pure culture. Environmental factors were pH (4, 6, 7, 8, 10), O_2_ concentrations (0, 0.1%, 1%, 5%, 21% v/v), NO_3_^-^ concentrations (0, 5, 50, 100, 500 mg/L), and carbon sources (Sodium acetate (SA), Glycerol, Disodium succinate (DS), Glucose, hydroxyethyl cellulose (HEC)). The data are expressed as the mean ± SEM for each treatment (n = 3).

The pure culture experiments demonstrated that environmental factors significantly influenced the growth and N_2_O-reducing activity of the *nosZ*I and *nosZ*II strains (**Figure 1**). One-way ANOVA revealed that the *nosZ*I strain exhibited optimal growth under acidic conditions (pH 4 and 6), while its highest N_2_O reduction efficiency was observed at neutral pH (pH 7), which differed significantly from other pH levels (p < 0.05). In contrast, the *nosZ*II strain thrived under alkaline (pH 8 and 10) and mildly acidic (pH 6) conditions, achieving peak N_2_O reduction at pH 6 and 8. Bivariate correlation analysis showed a significant negative correlation between pH and the growth of the *nosZ*I strain (r = -0.88, p < 0.01), and a significant positive correlation for the *nosZ*II strain (r = 0.78, p < 0.01).

Both strains grew well under fully aerobic conditions (21% O_2_). However, the *nosZ*I strain achieved its highest N_2_O reduction at 5% O_2_, while the *nosZ*II strain exhibited optimal N_2_O reduction under low-oxygen conditions (1% and 5% O_2_). A significant positive correlation between oxygen concentration and growth was observed only for the *nosZ*II strain (r = 0.84, p < 0.01).

Nitrate concentration had contrasting effects. The *nosZ*I strain grew best under low or absent nitrate, with maximum N_2_O reduction at 5 mg N L^-^¹. Higher nitrate levels led to reduced reduction efficiency and even net N_2_O emissions. Its growth was significantly negatively correlated with nitrate concentration (r = -0.75, p < 0.01), while N_2_O reduction was positively correlated with growth (r = 0.43, p < 0.05). Conversely, the *nosZ*II strain maintained stable growth across all nitrate levels, except under nitrate deficiency, where growth declined sharply. A strong positive correlation was observed between growth and N_2_O reduction for the *nosZ*II strain (r = 0.73, p < 0.01).

In terms of carbon sources, both strains exhibited optimal growth with glucose. The *nosZ*I strain also displayed its highest N_2_O reduction with glucose. However, the *nosZ*II strain achieved greater N_2_O reduction efficiency when sodium acetate (SA) or hydroxyethyl cellulose (HEC) served as the carbon source.

### 3.2 Soil N_2_O emissions with fertilization types and *nosZ*-clade type strains

**Figure 2:**
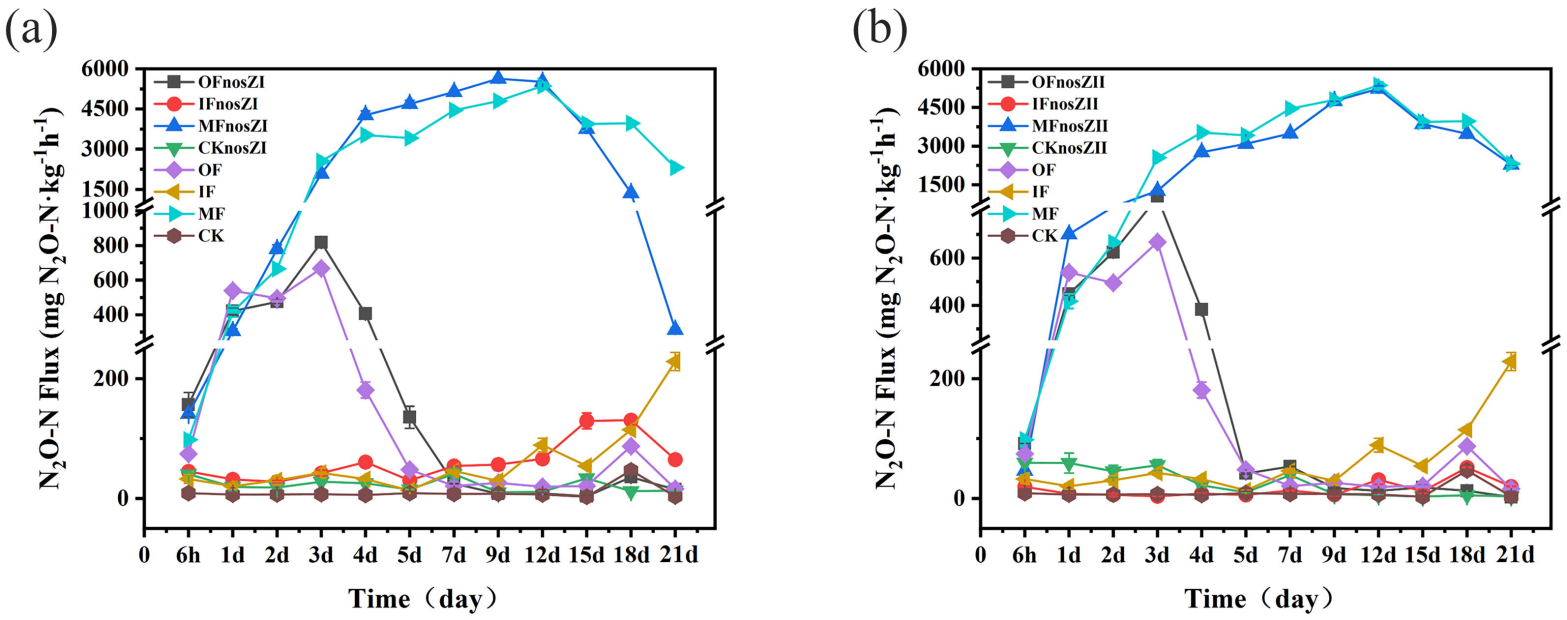
Soil N_2_O flux under different fertilization types inoculated with the *nosZ*I **(a)** or *nosZ*II **(b)** strain. OF represents organic fertilizer treatment; IF represents inorganic fertilizer treatment; MF represents mixed inorganic-organic fertilizer treatment; The suffix with *nosZ*I or *nosZ*II represent *nosZ*I or *nosZ*II strains adding treatments. CK represents control treatment without fertilization and strain. The data are expressed as the mean ± SEM for each treatment (n = 3).

N_2_O emission rates varied across treatments and over time (**Figure 2**). In soils treated with inorganic fertilizer (IF), emissions remained low for the first 9 days and then increased significantly after day 12, with no peak reached within the 21-day observation period. Soils treated with organic fertilizer (OF) exhibited peak emissions as early as day 4. In contrast, soils treated with a mixed fertilizer (MF) showed steadily increasing emissions, peaking on day 12. Repeated measures ANOVA showed that treatments inoculated with the *nosZ*II strain exhibited significantly lower N_2_O emission rates than their non-inoculated controls throughout the experiment (p < 0.01). However, this suppression effect was significant only in the IF*nosZ*II and MF*nosZ*II treatments. For *nosZ*I-amended soils, all treatments except IF*nosZ*I showed significantly altered emission rates compared to controls (p < 0.01), with emission suppression observed only in the MF*nosZ*I treatment. One-way ANOVA indicated that IF*nosZ*II (inorganic fertilizer + *nosZ*II) reduced N_2_O emissions by 55–91% during days 4–8 compared to IF alone (p < 0.05). The MF*nosZ*II treatment also significantly reduced emissions by 1.3–51% between days 3 and 21 (p < 0.05), although a slight increase in emissions was observed during the early incubation phase. In the MF*nosZ*I treatment, N_2_O emissions were reduced by 27–66% on days 1, 3, 15, 18, and 21 compared to MF (p < 0.05). At other time points, *nosZ*I either increased emissions or had no significant effect.

**Figure 3:**
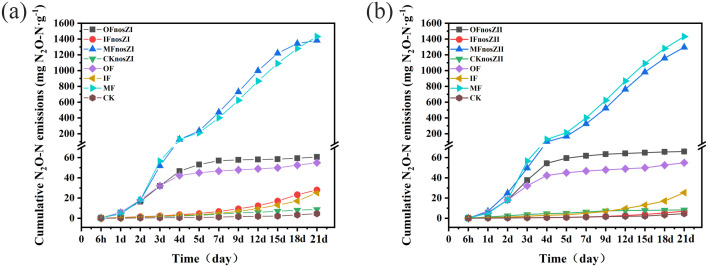
Cumulative N_2_O emissions under different fertilization types inoculated with the *nosZ*I **(a)** or *nosZ*II **(b)** strain. OF represents organic fertilizer treatment; IF represents inorganic fertilizer treatment; MF represents mixed inorganic-organic fertilizer treatment; The suffix with *nosZ*I or *nosZ*II represent *nosZ*I or *nosZ*II strains adding treatments. CK represents control treatment without fertilization and strain. The data are expressed as the mean ± SEM for each treatment (n = 3).

Cumulative N_2_O emissions across treatments are shown in **Figure 3**. One-way ANOVA revealed that fertilization type significantly influenced cumulative emissions (p < 0.05). Repeated measures ANOVA further confirmed that inoculation with *nosZ*I or *nosZ*II led to significant differences in cumulative emissions compared to uninoculated controls (p < 0.01), although a suppression effect was observed only in the IF*nosZ*II and MF*nosZ*II treatments. Cumulative emissions from IF, OF, and MF treatments were 25.32, 54.96, and 1431 mg kg^-^¹, respectively—representing 6-, 12-, and 312-fold increases relative to the CK control (4.59 mg kg^-^¹). In terms of reduction efficiency: IF*nosZ*II reduced cumulative N_2_O emissions by 72% (p < 0.01), MF*nosZ*II by 10% (p < 0.01), and MF*nosZ*I by 3.4% (p < 0.01), relative to their respective fertilized controls. All other inoculated treatments resulted in increased cumulative emissions. Specifically: OF*nosZ*II and OF*nosZ*I increased emissions by 20% and 10%, respectively, over the OF treatment. IF*nosZ*I increased emissions by 10% compared to IF. Two-way ANOVA revealed that fertilizer type, *nosZ* clade, and their interaction had significant effects on cumulative N_2_O emissions (p < 0.01). Notably, in the absence of fertilizer, inoculation with *nosZ*II or *nosZ*I resulted in 80% and 89% higher emissions, respectively, compared to the CK control.

## 4 Discussion

### 4.1 Responses of selected isolates to pH, O_2_, NO_3_^-^, and carbon source

Our findings demonstrate that *nosZ*II strains exhibit greater resilience in both growth and N_2_O-reducing activity under varying environmental conditions compared to *nosZ*I strains. Key environmental factors affecting *nosZ*-containing bacteria include pH, oxygen concentration, nitrate availability, and carbon source type.

Among these, pH is widely recognized as one of the most critical factors influencing microbial activity. Extreme pH conditions typically inhibit microbial metabolism, with most microbial inoculants favoring neutral to mildly acidic or alkaline environments. In our pure culture experiments, *nosZ*II strain growth was positively correlated with pH across a broad range (pH 4–10), while *nosZ*I strain growth showed a significant negative correlation. This indicates that *nosZ*II strains prefer neutral to alkaline conditions, whereas *nosZ*I strains perform better in neutral to acidic environments. Although *nosZ* enzyme activity is generally sensitive to both low pH and high salinity, previous studies have shown that certain strains—such as Rhodanobacter and Cloacibacterium—retain N_2_O reduction capability in low-pH environments (Lycus et al., 2017; Wang et al., 2023). Consistent with these findings, our *nosZ*II strain maintained high N_2_O-reduction activity across the entire tested pH range, suggesting it could be effective in both acidic and alkaline soils. In contrast, the *nosZ*I strain showed high activity only at neutral pH, with reduced performance under other conditions. The broader pH adaptability of the *nosZ*II strain may be due to enhanced enzyme efficiency and more robust stress response mechanisms.

Although both strains grew optimally under aerobic conditions, they were capable of reducing N_2_O under all tested O_2_ concentrations (0–21%), indicating a low dependence on oxygen. This trait suggests their potential effectiveness for field-level N_2_O mitigation under fluctuating oxygen conditions. Membrane-bound denitrification reductases are typically active under anaerobic conditions, while periplasmic reductases can function under both aerobic and anaerobic environments (Bell et al., 1990; Chen and Strous, 2013; Duan et al., 2015; Yang et al., 2020). Our findings suggest that both isolates may possess periplasmic nitrous oxide reductases, which would explain their flexibility under varying oxygen levels (Liu et al., 2023).

The two strains also exhibited contrasting responses to nitrate availability. The *nosZ*I strain performed best under low or absent NO_3_^-^, with high concentrations (100–500 mg/L) inhibiting its activity and even resulting in net N_2_O emissions. This may indicate an N_2_O/N_2_ production ratio greater than 1 due to partial denitrification or enzyme inhibition (Senbayram et al., 2019). In contrast, the *nosZ*II strain maintained stable growth and high N_2_O reduction efficiency across all NO_3_^-^ levels, including high concentrations, suggesting it is better adapted for nitrate-rich environments. These differences may be attributed to distinct enzymatic kinetics and substrate affinities between *nosZ*I and *nosZ*II nitrous oxide reductases.

Both strains showed optimal growth with glucose as a carbon source. However, *nosZ*II displayed the highest N_2_O reduction efficiency with sodium acetate, while *nosZ*I achieved peak efficiency with glucose and moderate efficiency with glycerol. The availability of different electron donors influences the structure of N_2_O-reducing microbial communities, often driving niche differentiation (Maheshwari et al., 2023). In our study, *nosZ*II demonstrated broader carbon source adaptability, maintaining consistently high N_2_O reduction across all tested sources, consistent with its widespread occurrence in diverse environments. This may reflect its capacity to metabolize a wider range of organic substrates, enhancing its survival and function under field conditions.

### 4.2 Soil N_2_O emissions and functional performance of selected isolates

In the soil microcosm experiment, the MF treatment (mixed fertilizer) produced significantly higher cumulative N_2_O emissions than the IF (inorganic), OF (organic), or CK (control) treatments (p < 0.01), aligning with previous studies (Feng et al., 2019; Sompouviset et al., 2023). This effect likely stems from the abundant labile carbon in composted sheep manure, which stimulates microbial activity, and the high nitrogen content in chemical fertilizers, both of which enhance denitrification and N_2_O emissions (Jassal et al., 2011; Senbayram et al., 2012; Montes et al., 2013; Zhou et al., 2017; Shakoor et al., 2021).

While some studies report N_2_O mitigation effects from organic fertilizers (Xie et al., 2024), our results showed that organic amendments led to higher emissions than inorganic ones, consistent with field trials in Northeast China (Liu et al., 2023). This discrepancy may stem from differences in soil properties, organic matter composition, microbial communities, and experimental conditions. For example, the C/N ratio of organic material is critical; cattle and pig manures often reduce N_2_O emissions, whereas rapeseed cake increases them (Zou et al., 2003; Zhang et al., 2020b).

Across treatments, the *nosZ*II strain consistently outperformed the *nosZ*I strain in terms of N_2_O reduction potential. Although both strains contained the *nosZ* and *nirK* genes, neither harbored *nirS*, indicating they can denitrify via the NO_2_^-^ → N_2_O → N_2_ pathway. However, in some inoculated treatments, N_2_O emissions increased, suggesting that *nosZ*-containing bacteria—particularly clade I—may contribute more to N_2_O production than reduction, depending on environmental context.

For example, in the IF and MF treatments inoculated with *nosZ*II, significant reductions in N_2_O emissions were observed, whereas in OF and CK treatments, emissions increased. These outcomes suggest that *nosZ*II strains are more effective in soils with high inorganic nitrogen, where nitrate levels may enhance their denitrifying capacity. Conversely, in low nitrate or high organic C/N environments, microbial N_2_O production may exceed reduction, resulting in net emissions. This aligns with our pure culture findings: *nosZ*I strain activity declined under increasing nitrate, while *nosZ*II activity remained high.

### 4.3 Practical implications, limitations and future perspectives

This study demonstrates the potential of II-containing bacteria for mitigating N_2_O emissions, especially in soils with high inorganic nitrogen content. The development of bio-organic fertilizers enriched with *nosZ*II strains represents a promising approach to reduce greenhouse gas emissions in agriculture, contributing to both climate mitigation and sustainable crop production.

Our study has several limitations. First, we used only two bacterial strains under a limited range of environmental conditions. Future research should expand the diversity of *nosZ*-containing isolates, examining their physiological traits and N_2_O-reducing capacities under field-relevant and extreme conditions. Second, future efforts should prioritize the isolation of strains containing *nosZ* independent of *nir* genes, to avoid potential N_2_O accumulation during partial denitrification. Such strains could serve as ideal candidates for bio-organic fertilizers aimed specifically at mitigating N_2_O emissions.

Based on our findings, we propose the following future directions: (1) Investigate the genetic and enzymatic differences between *nosZ*I and *nosZ*II strains that explain their divergent environmental responses. (2) Conduct field-scale experiments using diverse *nosZ*-containing isolates to evaluate their effectiveness in real agricultural systems. (3) Explore the development of bioformulations combining *nosZ*II strains with other beneficial microbes or soil amendments to enhance N_2_O mitigation while supporting soil health.

## 5 Conclusions

This study underscores the important role of *nosZ*-containing microbial isolates in regulating soil N_2_O emissions under different fertilization regimes. Our results show that the application of mixed organic and inorganic fertilizers significantly increases N_2_O emissions compared to single-source fertilizers, likely due to enhanced microbial activity driven by combined nutrient availability. Among the tested strains, the *nosZ*II isolate exhibited superior N_2_O-reducing capacity compared to the *nosZ*I isolate, particularly under conditions with elevated inorganic nitrogen. This highlights its potential as a targeted microbial agent for N_2_O mitigation in intensively managed agricultural soils. Environmental factors significantly influenced the performance of both strains. However, the *nosZ*II strain demonstrated greater resilience, showing broader pH tolerance, higher nitrate and oxygen tolerance, and greater adaptability to diverse carbon sources. While these findings offer promising insights for the development of microbial biocontrol strategies, the complexity of soil N_2_O dynamics warrants further investigation. Future research should prioritize the isolation of high-efficiency N_2_O-reducing strains that possess the *nosZ* gene independently of other denitrification genes (e.g., *nirS*, *nirK*), to minimize potential N_2_O production and enhance the reliability of microbial inoculants. Integrating these selected microbial strains into bio-organic fertilizers could serve as a practical and scalable solution for reducing N_2_O emissions from agriculture, contributing to both climate change mitigation and the advancement of sustainable agricultural practices.

## Data Availability

The raw data supporting the conclusions of this article will be made available by the authors, without undue reservation.

## Supplementary material

Supplementary Table 1 The amplification primers of PCR. Isolation and identification of nosZI or nosZII clade type strains.
