# Supplementary material for: Comparative study of nosZI and nosZII clade isolates: insights into their responses to environmental variables and soil fertilization types
Source: Front Plant Sci. 2025 Jun 6;16:1537010. doi: 10.3389/fpls.2025.1537010 (PMC12178887; doi:10.3389/fpls.2025.1537010)
Supplement: Supplementary Table 1 — The amplification primers of PCR. Isolation and identification of nosZI or nosZII clade type strains. [file Table1.docx]

Supplementary Material

# Isolation and identification of *nosZ*I or *nosZ*II clade type strains

Thirty grams of soil (oven-dry equivalent) was added to a pre-sterilized 500 ml Schott bottle, followed by the addition of sterile water until the water just covered the soil, maintaining anaerobic conditions. The soil was pre-incubated for 7 days, after which sterilized glass beads were added, and the mixture was shaken at 220 rpm for 5 minutes. After letting it stand for 5 minutes, 20 ml of the supernatant was transferred to another sterilized 500 ml Schott bottle. In this bottle, 0.3 g of sodium acetate anhydrous was added[1]. The headspace gas was replaced with high-purity helium (99.999%), and 40 ml of high-purity N_2_O (99.9%) was injected into the bottle with a syringe, bringing the N_2_O concentration to approximately 8%. The sample was incubated at 30°C and 180 rpm for enrichment, with the headspace gas replaced with helium and N_2_O (8%) every 2 days.

After 7 days of incubation, the liquid was diluted using a tenfold gradient with sterile water to obtain 10^-2^ to 10^-5^ dilutions. These dilutions were plated on nitrogen-free media, and the plates were placed in a sealed chamber with inlet and outlet ports. High-purity helium was flushed through the chamber to replace the air, and 50 ml of N_2_O (99.9%) was added. The plates were incubated at 30°C for 2 days. Different colonies with distinct morphology and color were selected and cultured in liquid nutrient media at 30°C and 180 rpm. Genomic DNA was extracted using a bacterial genomic DNA extraction kit (Biomed, China), and PCR amplification of *nosZ*I*,* and *nosZ*II was carried out according to the primers and conditions outlined in Table S1. The PCR reaction system contained 10 μL Premix Ex Taq (Takara Bio, China), 0.8 μM each primer, 1 μL genomic DNA template, and adding ddH_2_O up to 20μL. Several strains containing *nosZ*I or *nosZ*II gene were obtained. Finally, two strains with significant N_2_O emission reduction effects were selected as test strains through preliminary experiments involving pure cultivation in this study.

# Table S1. The amplification primers of PCR

| gene | primer | Primer sequence | RTFQ-PCR | Reference |
| --- | --- | --- | --- | --- |
| *nosZ*Ⅰ | NosZ1mod-R | ATRTCGATSARCTGVKCRTTYTC | 95℃,30s;95℃,10s;55℃,30s;72℃,40s;39 cycles | [2] |
|  | NosZ1mod-F | WCSYTSTTCMTSGAYAGCCAG |  |  |
| *nosZ*Ⅱ | NosZ-Ⅱc-R | GCGARCARAATTBGTRC | 95℃,30s;95℃,10s;54℃,30s;72℃,40s;39 cycles | [2] |
|  | NosZ-Ⅱb-F | CTGGCCYTMCAYAS |  |  |

[1] Maheshwari, A., Jones, C. M., Tiemann, M., Hallin, S. (2023). Carbon substrate selects for different lineages of N_2_O reducing communities in soils under anoxic conditions. *Soil Boil. Biochem.* 177. doi: 10.1016/j.soilbio.2022.108909

[2] Chee-Sanford, J. C., Connor, L., Krichels, A., Yang, W. H., Sanford, R. A. (2020). Hierarchical detection of diverse Clade II (atypical) *nosZ* genes using new primer sets for classical- and multiplex PCR array applications. *J. Microbiol. Methods*. 172, 105908. doi: 10.1016/j.mimet.2020.105908
